# Palestinian pharmacists’ knowledge of issues related to using psychotropic medications in older people: a cross-sectional study

**DOI:** 10.3352/jeehp.2017.14.8

**Published:** 2017-04-24

**Authors:** Ramzi Shawahna, Mais Khaskiyyi, Hadeel Abdo, Yasmen Msarwe, Rania Odeh, Souad Salame

**Affiliations:** 1 Department of Physiology, Pharmacology and Toxicology, Faculty of Medicine and Health Sciences, An-Najah National University, Nablus, Palestine; 2 An-Najah BioSciences Unit, Centre for Poisons Control, Chemical and Biological Analyses, An-Najah National University, Nablus, Palestine; 3 Department of Pharmacy, Faculty of Medicine and Health Sciences, An-Najah National University, Nablus, Palestine; Hallym University, Korea

**Keywords:** Cross-sectional studies, Geriatrics, Knowledge, Pharmacists, Psychotropic drugs

## Abstract

**Purpose:**

The purpose of this study was to assess the knowledge of pharmacists practicing in Palestine of issues related to using psychotropic medications in older people.

**Methods:**

The study was conducted with a cross-sectional observational design using a questionnaire. A total of 400 pharmacists responded to a 19-statement knowledge test related to the use of psychotropic medications in older people. The study was conducted from July 2016 to February 2017. The reliability and internal consistency of the study tool was assessed using the test-retest method and the Cronbach alpha. Categorical groups were compared using the chi-square test and the Spearman rank correlation.

**Results:**

On the 19-statement knowledge test, the median score was 55.3% with an interquartile range of 21.9%. In a comparison of the demographic and practice-related variables of the pharmacists who scored ≥ 50% on the 19-statement knowledge test with those who scored < 50%, age, gender, and having taken a course on psychotropic medications were found to be significantly associated with performance, as shown by the chi-square test and Spearman correlation.

**Conclusion:**

Pharmacists practicing in Palestine possess less than optimal knowledge of issues related to the use of psychotropic medications in older people. Continuing educational interventions and/or training might be helpful in improving pharmacists’ knowledge of issues related to using psychotropic medications in older people.

## Introduction

The provision of healthcare services has reduced mortality rates and increased life expectancy around the world. Consequently, the world’s population is aging and the number of older people is increasing dramatically. Older people often suffer chronic mental disorders and consequently utilize psychotropic medications. Recently, the utilization of psychotropic medications by older people was reported to be increasing [1]. Pharmacists are trusted and easily accessible healthcare professionals who are expected to provide healthcare for patients, including older people, who use psychotropic medications. As experts in medication use, pharmacists can play an indispensable role in caring for patients, including older people, promoting the rational use of psychotropic medications, and helping patients obtain the best benefits from their medications. To provide optimal healthcare services, pharmacists are expected to have an adequate knowledge of medications. Assessing pharmacists’ knowledge of various aspects of medications could be a part of licensing examinations and can serve as a quality measure in practice [2-4]. The purpose of this study was to assess the knowledge of pharmacists practicing in Palestine of issues related to using psychotropic medications in older people.

## Methods

### Study design

The study was conducted among practicing Palestinian pharmacists with a cross-sectional observational design using a questionnaire. The study questionnaire was based on previous studies conducted elsewhere [5,6]. Participants responded to a 19-statement knowledgetest related to psychotropic medication use in older people. Respondents had to choose either ‘true,’ ‘false,’ or ‘I don’t know’ for each item. Respondents were awarded one point for each correct answer and zero point for choosing the ‘I don’t know’ option. If the respondent selected the wrong answer, half a point was deducted as a penalty for guessing [7]. Final scores were calculated for the 19-statement knowledge test as the percentage correct, which ranged from 0% to 100%.

Demographic and practice-related details such as age, gender, place of residence, academic degrees, number of years in practice, and practice settings were also collected from each participant. Additionally, participants were asked if they had taken a course on psychotropic medications during their pharmacy degree programs. The study participants completed the questionnaire in privacy in their workplaces in approximately 25 minutes. This study was conducted without any financial incentives.

### Study subjects

The sample size required for this study was estimated using a sample size calculator (www.raosoft.com). The estimated population of pharmacists in Palestine is approximately 6,000. Using a confidence interval (CI) of 95% and a margin of error of 5%, a sample size of about 360 pharmacists was required for this study. In this study, 500 pharmacists were approached and invited to respond to the questionnaire. The inclusion criteria were as follows: (1) having a basic or advanced degree in pharmacy, (2) being licensed to practice in Palestine, and (3) being willing to complete the study questionnaire. Pharmacy assistants and trainees were excluded. Pharmacists were visited in their workplaces by field researchers who explained the study objectives to potential participants and obtained verbal consent. In Palestine, a pharmacist can obtain a license to practice pharmacy after completing either a bachelor’s (BSc) degree in pharmacy or a doctor of pharmacy (PharmD) degree and passing a licensing examination [7]. To ensure representativeness, pharmacists were recruited from different regions of the West Bank of Palestine. A convenience sampling technique was used to recruit the sample required for this study. The study was conducted from July 2016 to February 2017.

### Reliability of the study questionnaire

To test the stability of scores over a short period of time, the test-retest method was used. Questionnaires were completed by a group of 25 pharmacy graduates twice after allowing a time interval of 30 minutes to 1 hour between each administration. Correlations among the score percentages obtained by the same persons in both administrations were evaluated using Pearson correlation analysis. As in previous studies, a Pearson correlation coefficient of >0.80 was set a priori as indicative of acceptable test-retest reliability [4,7,8]. The Cronbach alpha was used to ensure that the questionnaire was internally consistent. Cronbach alpha values in the range of 0.70–0.95 were considered acceptable.

### Statistics

The statistical analysis was performed using IBM SPSS for Windows ver. 21.0 (IBM Corp., Armonk, NY, USA). The normality of the distribution was assessed using the Kolmogorov-Smirnov test. As the data were not normally distributed, medians were used, with the lower (Q1) and upper quartiles (Q3). To calculate the interquartile range (IQR), Q1 was subtracted from Q3. Pharmacists were considered to have passed the 19-statement knowledge test if they scored ≥ 50%. The Pearson chi-square test was used to compare categorical groups. The Spearman rank correlation was used to correlate variables with score percentages. P-values ≤ 0.05 were considered to indicate statistical significance.

### Ethical approval

This study was reviewed and approved by the institutional review board of An-Najah National University (IRB approval no. May-27-2016).

## Results

The test-retest reliability of the questionnaire was excellent, as indicated by a Pearson’s correlation coefficient of 0.95 (95% CI, 0.90 to 0.97) with a P-value of < 0.01. The Cronbach alpha of this questionnaire was 0.74, indicating good internal consistency of the items used. Raw data were available from Supplement 1.

The questionnaire was completed by 400 of the 500 pharmacists initially invited, giving a response rate of 80%. The demographic and practice details of the pharmacists who took part in this study are shown in Table 1.

A total of 330 (82.5%) of the pharmacists who participated in this study were younger than 35 years old and 201 (50.3%) were men. The vast majority of them had a basic degree in pharmacy, and 234 (58.5%) of the pharmacists stated that they had taken a course on psychotropic medications during their pharmacy degree program (Table 1).

On the 19-statement knowledge test, the median score was 55.3%, with an IQR of 21.9%. On some questions, a considerable percentage of the pharmacists responded with ‘I don’t know’ instead of guessing the answer incorrectly. Details of the response distribution for each item of the 19-statement knowledge test are shown in Table 2.

In a comparison of the demographic and practice-related variables of the pharmacists who scored ≥ 50% on the 19-statement knowledge test with those who scored <50%, age, gender, and having taken a course on psychotropic medications were found to be significantly associated with performance, as shown by the chi-square test and Spearman correlation analysis. The associations of the demographic and practice-related variables with knowledge are shown in Table 3.

Other demographic and practice-related variables, such as place of residence, academic degrees, number of years in practice, and practice setting, were not significantly associated with knowledge (Table 3).

## Discussion

In this study, pharmacists’ knowledge of issues related to pharmacotherapy using psychotropic medications in older people was assessed. Our results extend previous assessments of pharmacists’ knowledge of issues related to excipients, the pharmacotherapy of epilepsy, women’s issues in epilepsy, and autism [3,4,7,8]. To the best of our knowledge, this study is the first assessment of pharmacists’ knowledge of psychotropic medication use in older people in Palestinian pharmacy practice. In this study, a reliable and internally consistent tool was used. Our results showed that the statements included in the knowledge test varied in the level of difficulty, as shown by the answers of the participants. The sample included participants from both genders and different age groups, geographical locations, experience, and practice settings (Table 1). This ensured representativeness of the community of Palestinian pharmacists.

Pharmacists are trained as experts in medications, and in modern healthcare systems they are indispensable sources of information who are supposed to help patients make better use of their medications. To do so, pharmacists should be knowledgeable about issues related to pharmacotherapy; in our study, this was assessed in terms of using psychotropic medications in older people. Although the median score on the 19-statement knowledge test used in this study was modest (55.3% with an IQR of 21.9%), some areas of good knowledge can be highlighted.

When asked about the dosage of psychotropic medications (Table 2, statements 1–4), 286 (71.5%) of the pharmacists knew that the dose of antipsychotics or benzodiazepines should be reduced in older people due to altered metabolism rate and higher sensitivity. Of the pharmacists, 279 (69.8%) knew the recommended daily dose of risperidone in older people with severe behavioral disorders in dementia, and a similar percentage knew that low-dose hypnosedative medications given over the short term might reverse the physiological changes in sleep patterns in older people. However, only 159 (39.8%) could correctly answer the statement on the recommended daily dosage of olanzapine in older people with severe behavioral disorders in dementia. In general, the pharmacists surveyed in this study gave more correct answers than the nurses in the study of Wauters et al. [6] in Belgium. Today, the role of the pharmacist goes beyond merely dispensing medications. Pharmacists can play an important role in helping physicians select the appropriate dosage, as it has been reported that pharmacist-directed interventions promoted dose optimization [9,10]. However, lack of knowledge can limit the potential role of pharmacists in caring for patients and might jeopardize the health of their patients.

The performance of the pharmacists on statements related to the selection of appropriate psychotropic medications varied by statement (Table 3, statements 5–11). Of the pharmacists surveyed, 339 (84.8%) knew that when non-pharmacological therapies fail, hypnosedatives are to be used for the treatment and minimization of anxiety disorders, 298 (74.5%) knew that antipsychotics can have a place in the treatment of delirium, 294 (73.5%) knew that hypnosedatives should be administered for a short period of time in old people, 288 (72%) knew that antipsychotics reduce delusions and hallucinations, 256 (64%) knew that antipsychotics are preferred to benzodiazepines for sedating older patients with severe agitation or delirium, 222 (55.5%) knew that antipsychotic medications are preferred over behavior-oriented therapy in older people with dementia, and 212 (53%) knew that diazepam is not appropriate for use in older people. Again, the pharmacists in this study performed better than the Belgian nurses in the study of Wauters et al. [6]. Pharmacy as a profession is evolving. Pharmacists are often consulted by physicians in selecting the most appropriate medications for patients [9]. Therefore, the knowledge of pharmacists should be optimized. Failing to help physicians choose the appropriate psychotropic medication for an older patient might have severe negative consequences on the health of the patient concerned. Many pharmacy schools have realized this and revised their pharmacy curricula accordingly, in an attempt to acquaint pharmacy graduates with an optimal level of knowledge [11].

In this study, the performance of pharmacists on statements related to the dosage and selection of the appropriate psychotropic medications was better than their performance on statements related to the side effects of psychotropic medications (Table 3, statements 13– 19). Of the pharmacists, 334 (83.5%) knew that antipsychotics are associated with anticholinergic side effects, 314 (78.5%) knew that hypnosedatives can lead to dependence, 303 (75.8%) knew that benzodiazepines impair memory in older people, 268 (67%) knew that atypical antipsychotics can lead to weight gain, 251 (62.8%) knew that haloperidol was associated with akathisia and antipsychotics increased the prevalence of falls, 237 (59.3%) knew that antipsychotics cause postural hypotension, and 217 (54.3%) knew that antipsychotics increase the risk of cerebrovascular accidents. Despite gaps in knowledge, again, pharmacists in this study showed better knowledge than those reported in nurses in the study of Wauters et al. [6]. As experts on medications, pharmacists need to be knowledgeable of side effects and drug-drug interactions, including those of psychotropic medications. If well acquainted, pharmacists can play a prominent role in resolving medication-related problems [9]. Pharmacists’ inability to alert patients as well as their physicians of the potential side effects of psychotropic medications might severely impact the health of the patients and might limit the role that pharmacists could play in caring for patients.

Interestingly in this study, younger pharmacists had better knowledge than their older counterparts (Table 3). This could be attributed to the inclusion of more information about psychotropic medications in current pharmacy curricula [11]. Female pharmacists also performed better than their male peers. In previous studies, gender was shown to be associated with knowledge among pharmacy students. Contrary to our findings, Umair Khan et al. [12] showed that male pharmacy students were more knowledgeable than their female peers about the side effects of medications. In the same study, pharmacy students were more knowledgeable of side effects of medications and reported more positive attitudes regarding their capacity to handle and report these side effects than medical students. In this study, taking a course on psychotropic medications during the pharmacy degree program was significantly associated with scoring 50% or above on the knowledge test (Table 3). Our results were concordant with those previously reported among pharmacists in Palestine [4]. Pharmacists who had taken courses on antiepileptic medications were more knowledge of issues in the pharmacotherapy of epilepsy. Designing courses on psychotropic medications and incorporating issues related to the use of these medications in older people might enhance pharmacists’ knowledge. Incorporating practical sessions in pharmacology and pharmacotherapy might have positive effects on the knowledge, attitudes, and skills of pharmacists [13]. Recently, it has been reported that practical training programs increased learning motivation among pharmacy students [14]. Similarly, using computer simulations was shown to enhance knowledge of medications and pharmacology [15].

Our results should be interpreted with some limitations in mind. First, a convenience sampling strategy was used. However, a representative sample was included in this study, and a sample size calculator was used to determine the sample size. The sample included participants from both genders, different age groups, locations, and practice settings, which might have promoted representativeness and reduced bias. Second, respondents had to choose ‘true,’ false,’ or ‘I don’t know’ for each test item. The use of this tool could have underestimated or overestimated the knowledge of pharmacists, as their performance could have been different if the test was based on multiple-choice questions [3]. Third, the test included questions in the form of statements. Respondents’ performance could have been different if case-based scenarios were included in the test [4]. However, the study tool used in this study was previously used in another setting [6]. Again, the tool was tested for test-retest reliability and internal consistency before the study was conducted. Finally, we deducted half a point for each wrong answer. This could be viewed as conservative and might have resulted in underestimating pharmacists’ knowledge of psychotropic medications.

In conclusion, our findings suggest that despite some areas of good knowledge, pharmacists possess less than optimal knowledge in issues related to the use of psychotropic medications in older people. Pharmacists who were females, less than 35 years of age, and had taken a course on psychotropic medications during their pharmacy degree program possessed better knowledge that their male, older peers who had not taken a course on psychotropic medications during their pharmacy degree program. Future studies should explore the potential of continuing education or training interventions to improve knowledge about psychotropic medication use in older people.

## Figures and Tables

**Table 1.** Demographic details of the pharmacists in this study

Variable	No. (%)
Age (yr)	
<35	330 (82.5)
≥35	70 (17.5)
Gender	
Male	201 (50.3)
Female	199 (49.8)
Place of residence	
Urban	290 (72.5)
Rural	110 (27.5)
Degree	
Basic degree (BSc Pharm or PharmD)	395 (98.8)
Higher degree (MSc or PhD)	5 (1.2)
No. of years in practice (yr)	
<5	208 (52.0)
≥5	192 (48.0)
Practice setting	
Community pharmacy	288 (72.0)
Hospital pharmacy	102 (25.5)
Others (insurance, etc.)	10 (2.5)
Had taken a course on psychotropic medications during their	
pharmacy degree program	
Yes	234 (58.5)
No	166 (41.5)

BSc Pharm, bachelor of science in pharmacy; MSc, master of science in pharmacy; PharmD, doctor of pharmacy; PhD, doctor of philosophy

**Table 2.** Test of pharmacists’ knowledge of psychotropic medication use in geriatric patients

Statement	Response distribution				
	True	False	I don’t know		
Statements related to dosage			
1.Due to changed metabolism and a higher sensitivity, older persons need a lower dose of antipsychotics or benzodiazepines in order to get the same effect.	286 (71.5)	87 (21.8)	27 (6.8)
2.The recommended daily dosage of olanzapine (Zyprexa) is 50 to 100 mg in older people with severe behavioral disorders in dementia.	175 (43.8)	159 (39.8)	66 (16.5)
3.The recommended daily dose of risperidone (Risperdal) is 0.5 to 2 mg in older people with severe behavioral disorders in dementia.	279 (69.8)	85 (21.3)	36 (9.0)
4.Ageing is associated with physiological changes in the sleeping pattern. To reverse these changes in the sleep pattern, hypnosedative medications may be used in low dose during a short period.	279 (69.8)	89 (22.3)	32 (8.0)
Statements related to selection of appropriate medications			
5.With the exception of delirium tremens, antipsychotics are preferred above benzodiazepines for sedating older patients with severe agitation or delirium.	256 (64.0)	81 (20.3)	63 (15.8)
6.Antipsychotic medications can have a place in the treatment of delirium.	298 (74.5)	66 (16.5)	36 (9.0)
7.Antipsychotic medications reduce such symptoms as delusions and hallucinations.	288 (72.0)	82 (20.5)	30 (7.5)
8.In the care of older adults with dementia, antipsychotic medications are preferred over behavior-oriented therapy.	222 (55.5)	134 (33.5)	44 (11.0)
9.Only in severe cases of sleeplessness and failure of alternative therapies with proven effectiveness, hypnosedatives can be administrated for a short period of time in the old.	294 (73.5)	76 (19.0)	30 (7.5)
10.The effects of diazepam (Valium), a benzodiazepine, can last for a long time (up to 300 hours), making it not proper to use in this age category.	212 (53.0)	112 (28.0)	76 (19.0)
11.Next to non-pharmacological therapies, hypnosedatives are to be used for treatment and minimization of the symptoms of anxiety disorders.	339 (84.8)	36 (9.0)	25 (6.3)
Statements related to side effects			
12.Use of hypnosedatives can lead to physical and emotional dependency.	314 (78.5)	61 (15.3)	25 (6.3)
13.Long-term (3 months or above) intake of antipsychotic medications increases the risk for cerebrovascular accidents.	217 (54.3)	126 (31.5)	57 (14.3)
14.Antipsychotic medications can cause side effects in the old such as disorientation, urine retention, dry mouth, and blurred vision.	334 (83.5)	43 (10.8)	23 (5.8)
15.Long-term intake (3 months or above) of atypical antipsychotic medications can lead to an increase in weight.	268 (67.0)	81 (20.3)	51 (12.8)
16.Patients starting on antipsychotic medication are susceptible to postural hypotension.	237 (59.3)	114 (28.5)	49 (12.3)
17.One of the side effects of Haloperidol (Haldol) is akathisia, which manifests with constant pacing and restlessness.	250 (62.5)	89 (22.3)	61 (15.3)
18.There is a connection between long-term (3 months or above) intake of antipsychotic medications and the prevalence of falls in the old.	251 (62.8)	67 (16.8)	82 (20.5)
19.Benzodiazepines can lead to side effects in the old like confusion, memory and concentration disorders.	303 (75.8)	62 (15.5)	35 (8.8)

Values are presented as number (%). Correct answers are in boldface.

**Table 3.** Association between pharmacist’s characteristics and scoring ≥ 50% on the 19-statement knowledge test

Variable	No. (%)	< 50%	≥ 50%	x^2^	P-value	Correlation	P-value
Age (yr)				4.04	0.044	-0.10	0.045
<35	330 (82.5)	92	238				
≥35	70 (17.5)	28	42				
Gender				4.48	0.04	0.11	0.034
Male	201 (50.3)	70	131				
Female	199 (49.8)	50	149				
Place of residence				1.49	0.271	0.07	0.163
Urban	290 (72.5)	82	208				
Rural	110 (27.5)	38	72				
Degree				0.65	0.654	0.07	0.140
Basic degree (BSc Pharm or Pharm的	395 (98.8)	229	166				
Higher degree (MSc or PhD)	5 (1.2)	2	3				
No. of years in practice (yr)				0.92	0.382	-0.05	0.338
<5	208 (52.0)	58	150				
≥5	192 (48.0)	62	130				
Practice setting				0.64	0.756	-0.06	0.223
Community pharmacy	288 (72.0)	165	123				
Hospital pharmacy	102 (25.5)	59	43				
Others (hospital, insurance)	10 (2.5)	7	3				
Had a course on psychotropic medications during their pharmacy degree program				4.15	0.047	-0.10	0.042
Yes	234 (58.5)	61	173				
No	166 (41.5)	59	107				

BSc Pharm, bachelor of science in pharmacy; MSc, master of science in pharmacy; PharmD, doctor of pharmacy; PhD, doctor of philosophy.

## Supplementary material

Supplement 1. Data file is available from https://dataverse.harvard.edu/dataset.xhtml?persistentId=doi:10.7910/DVN/ARRAGK

Supplement 2. Audio recording of the abstract.
